# Vaccination against *Staphylococcus aureus* mastitis in two Swedish dairy herds

**DOI:** 10.1186/s13028-015-0171-6

**Published:** 2015-11-25

**Authors:** Håkan Landin, Marie Jansson Mörk, Maria Larsson, Karin Persson Waller

**Affiliations:** Växa Sverige, SE-104 25 Stockholm, Sweden; Evidensia Djurkliniken Näset, SE-236 32 Höllviken, Sweden; Department of Animal Health and Antimicrobial Strategies, National Veterinary Institute (SVA), SE-75189 Uppsala, Sweden; Department of Clinical Sciences, Swedish University of Agricultural Sciences, Uppsala, Sweden

**Keywords:** Dairy cow, Mastitis, *Staphylococcus aureus*, Vaccine

## Abstract

**Background:**

*Staphylococcus aureus* is a common udder pathogen in dairy cows, and may cause severe mastitis problems in some herds. In herds where normal control measures are not successful, vaccination might be an additional tool to use if sufficiently efficient. The aim of the present study was to evaluate the efficacy of a commercially available vaccine (Startvac^®^, Hipra, Spain) in two commercial Swedish dairy herds where the control programs for *S. aureus* mastitis had been unsuccessful. Within each herd cows were randomly assigned to vaccine or control groups, and effects on udder health and milk production during 120 days after calving, and survival during the following lactation were evaluated.

**Results:**

A field study was performed in two high producing Swedish herds having approximately 600 (herd A) and 200 (herd B) cows. During 12 months, cows with odd numbers were vaccinated three times around calving according to label protocol, while cows with even numbers constituted the not vaccinated control group. Quarter milk samples for bacteriological culturing were collected from all cases of clinical and subclinical mastitis. The outcome was evaluated during 120 days after calving using data on SCC and daily milk yield at monthly milk recordings, and incidence of mastitis due to *S. aureus*, coagulase-negative staphylococci, streptococci and coliforms. Cow survival throughout lactation was also studied. In herd A, 239 and 240 cows were included in the vaccinated and control groups, respectively. Corresponding numbers for herd B was 126 and 151 cows. Significant differences between vaccinated and control groups were not found in any of the parameters investigated.

**Conclusions:**

Vaccination with a commercial polyvalent vaccine did not have any beneficial effects on udder health, milk production or survival in two commercial dairy herds with mastitis problems due to *S. aureus*.

## Background

*Staphylococcus aureus* is the most common bacterial finding in both subclinical (SCM) and clinical (CM) mastitis in Swedish dairy cows [1, 2] as well as in dairy cows in many other countries [3, 4]. Thus, such udder infections are of substantial economic importance for the dairy industry.

Common control measures for *S.aureus* mastitis are identification, segregation, treatment and culling of infected cows as well as improvements of important management routines such as milking hygiene [3, 4]. In some herds, these control measures have, however, not been able to successfully prevent spread of infections between cows. Therefore, other control measures, such as vaccines, are sometimes warranted.

As reviewed by Pereira et al. [5], many vaccines against intra-mammary infections (IMI) and mastitis due to *S. aureus* have been tested, but the results have been varying, especially when testing the vaccines in field studies. Moreover, few *S. aureus* vaccines have been commercialized, and none have been available within the EU until relatively recently when a new vaccine (Startvac^®^, Laboratorios Hipra, Spain) directed against *S. aureus,* coagulase-negative staphylococci (CNS) and *Escherichia coli* was introduced. This vaccine is also available in some of the Nordic countries. The staphylococcal component of the vaccine is a bacterin based on a *S. aureus* strain with the ability to express a slime-associated antigen complex (SAAC) involved in biofilm production [6, 7]. According to registration information [8], the vaccine reduce the incidence of *S. aureus* IMI, and the severity of clinical signs, but at the start of the present study no studies on efficacy of the vaccine in production herds using random selection of cows into equally sized experimental and control groups, had been published in scientific journals. Recently, a few field studies have, however, been published [9, 10]. Those studies, as well as the one included in the vaccine registration files, were performed in countries where the herd structure, production level and udder health is different from the Swedish situation. Moreover, *S. aureus* strains may differ between countries and regions [11, 12], and in their ability to produce SAAC and biofilm [6, 7]. When testing almost 300 *S. aureus* isolates collected in Swedish surveys on CM and SCM only 31 % of the isolates were positive in the Congo red agar (CRA) plate test which indicates slime production (K. Persson Waller, unpublished data). When evaluating vaccine efficacy, long-term effects on milk somatic cell count (SCC) and milk production are of substantial interest for the farmer. Only one [10] of the studies mentioned above include information on such data.

The aim of the present study was to evaluate the efficacy of a commercially available vaccine (Startvac^®^) on *S. aureus* mastitis in two commercial Swedish dairy herds where the control programs for *S. aureus* mastitis had been unsuccessful. Within each herd cows were randomly assigned to vaccine or control groups, and the outcome was evaluated using data on milk production, udder health (cow composite milk SCC, SCM, and mastitis cases with growth of *S. aureus*, CNS, streptococci or coliforms), and survival.

## Methods

### Herds

Two dairy herds (A and B) with *S. aureus* mastitis problems for at least 5 years according to herd veterinarians and owners were enrolled in the study. Traditional control measures consisting of identification and segregation of infected cows, culling of chronically infected cows, selective dry cow therapy, control of milking equipment and improvement of hygienic measures had been performed but were considered unsuccessful.

Both herds were situated in the southern one-third of Sweden, had warm free-stall housing systems and were enrolled in the Swedish Official Milk Recording Scheme (SOMRS, Växa Sverige, Stockholm, Sweden). Herd A milked their cows in a milking parlor, while herd B used a milking rotary. Information on numbers of cows, milk production, bulk milk SCC, and proportion of cows veterinary-treated for clinical mastitis (VTCM) the year before the start of the study are given in Table 1. In herd A, the distribution of breeds among all cows in the herd was 21 % Swedish Red (SR), 72 % Swedish Holstein (SH) and 7 % other breeds, while the corresponding distribution in herd B was 63 % SR, 35 % SH and 2 % other breeds.

**Table 1:** Descriptive statistics of herd data for the two herds included in a study on vaccination with Startvac^®^ for the 12-months period preceding the start of the trial

Herd data	Herd A	Herd B
Number of cows	628	172
Average milk yield, kg ECM/cow/year	11,546	11,085
Arithmetic average bulk milk SCC/ml	293,000	160,000
Proportion of cows veterinary-treated for clinical mastitis (%)	17	28

### Study design including farm data registration and milk sampling

Vaccinations were performed during a 12-months period starting in October 2010. Cows with uneven eartag numbers were vaccinated three times according to the manufacturer’s instructions, i.e. 45 days before expected calving, 35 days after the first vaccination, and 62 days after the second vaccination. Cows with even eartag numbers were not vaccinated and constituted the control group. For practical reasons a deviation from the vaccination protocol was done in herd A, the first vaccination was performed during the period 45–60 days before expected calving. On both herds vaccination was performed on one or two specific weekdays for practical reasons.

All heifers and cows present in the herds 6 months before the start of the trial were included in the study. The farmers registered all animals in an Excel document at the latest 45 days before expected calving. Information on group (vaccinated or control), expected calving day, day of actual calving and milk SCC at the first 4 monthly milk recordings after calving was registered for each cow. When occurring, day of VTCM, and day and cause of culling, was also registered. The file was sent every 3 months to the first author via e-mail. From all cows included in the study aseptic quarter milk samples were taken from affected udder quarters when CM or SCM were suspected during the first 4 months after calving. The samples were sent the same day via postal mail to the National Veterinary Institute (SVA), Uppsala. After culturing and identification of bacterial growth according to accredited routines at the laboratory, the results were registered in the SOMRS database.

### Collection of additional data

For cows included in the study, individual cow data (such as breed, lactation number, calving dates, genetic merit for milk production, monthly milk recording data on daily milk yield and SCC, culling, results from culturing of milk samples) from 6 months before the start of vaccination until 1 year after the end of the vaccination period (November 2012) was collected from the SOMRS.

### Data editing and statistical analyses

Data editing and statistical analyses were performed using Stata (Stata Statistical Software: Release 9.2; College Station, TX, USA: StataCorp LP).

The effects of vaccination on milk production, udder health [cow composite milk SCC, SCM, and mastitis cases with specific growth of *S. aureus*, CNS, streptococci or coliforms (*Escherichia coli*, *Klebsiella* spp)] and survival (slaughter, death) were evaluated. For milk production (kg milk/day) and SCC, data from the first 4 monthly milk recordings after calving were used. The occurrence of SCM was defined as SCC ≥ 200,000 cells/ml at any of the first 4 monthly milk recordings. Only cows with SCC data for all four milk recordings were used. Growth of the above-mentioned specific udder pathogens in tested milk samples originating in udder quarters with CM or SCM was registered when at least one milk sample with growth of one udder pathogen was found during the follow-up period.

Differences in SCC and milk production between vaccinated and unvaccinated cows at the first four milk recordings were analyzed using mixed-effects linear regression models, including cow identity as random effect. SCC and milk production was transformed using the natural logarithm. The effect of vaccination on the prevalence of SCM and mastitis due to *S. aureus*, CNS, streptococci or coliforms was analyzed using logistic regression models. Two models for SCM were run; (1) including all cows, and (2) only including primiparous cows and multiparous cows without SCM before dry-off (new SCM). The effect of vaccination on survival was analyzed using survival analysis (Cox proportional hazard models). The number of days from calving to exit from the herd (culling/death/euthanasia due to udder disease) was calculated. A cow was right censored if she had not calved again, was culled due to other reasons than udder disease or was still in the herd at the end of the follow-up period.

In all models vaccination (yes/no), breed (SR, SH, other), parity (1, 2, ≥3), SCM status at dry-off (1st parity and healthy, 2nd parity and healthy, 2nd parity and SCM, ≥3rd parity and healthy, ≥3rd parity and SCM), herd (A, B), and proportion of cows vaccinated (<10, 10–19, 20–29, 30–39, ≥40 %) were included as explanatory variables. In the analyses of SCC and milk production days in milk (DIM) were included as a categorical fixed effect (categorized into quartiles).

The fit of the linear regression models was evaluated by visual inspection of plots of standardized residuals vs predicted values, and Q–Q plot of standardized residuals. The fit of the logistic regression models and Cox proportional hazard models was evaluated by goodness-of-fit tests.

## Results

In total, data was available for 614 cows (620 lactations) in herd A, and 316 cows (323 lactations) in herd B. Of those, 135 and 39 cows were excluded in herd A and B, respectively. Reasons for exclusion were that cows were dried off early, wrongly vaccinated or not vaccinated, culled or died, forgotten, and dried off before slaughter, or had missing data on vaccination date, could not be caught on pasture, were not pregnant/had aborted, and calved earlier than planned. Further details on vaccinated and unvaccinated cows are given in Table 2. At the time of calving for the first vaccinated cow, 25 and 15 cows had started their vaccination scheme in herd A and B, respectively.

**Table 2:** Descriptive statistics on cows included in a study on vaccination with Startvac^®^ in two herds

Cow data	Herd A		Herd B	
	Vaccinated	Unvaccinated	Vaccinated	Unvaccinated
Cows, n	239	240	126	151
Lactations, n	245	240	129	155
SR, n (%)	106 (21.9)		208 (73.2)	
SH, n (%)	360 (74.2)		71 (25.0)	
SR*SH,	17 (3.5)		3 (1.1)	
Other, n (%)	2 (0.4)		2 (0.7)	
Primiparous, n (%)	219 (45.3)		101 (35.6)	
Cows at 1st milk recording	463		281	
Cows at 2nd milk recording	458		280	
Cows at 3rd milk recording	449		275	
Cows at 4th milk recording	438		268	

*SR* Swedish Red, *SH* Swedish Holstein, *Other* other breeds and crosses

### SCC and milk production at monthly milk recordings

**Fig. 1:**
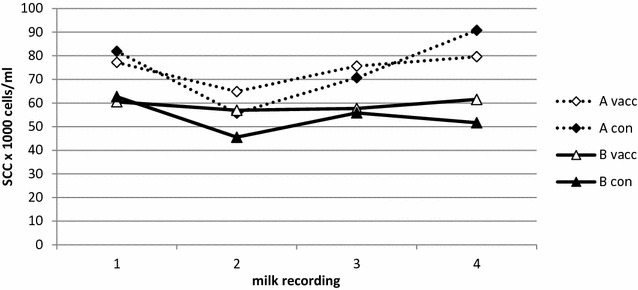
Somatic cell counts in vaccinated and not vaccinated cows. Cow composite milk somatic cell count (SCC; geometric average) at the first 4 monthly milk recordings after calving in vaccinated (vacc) and not vaccinated control (con) cows in herd A and B

**Fig. 2:**
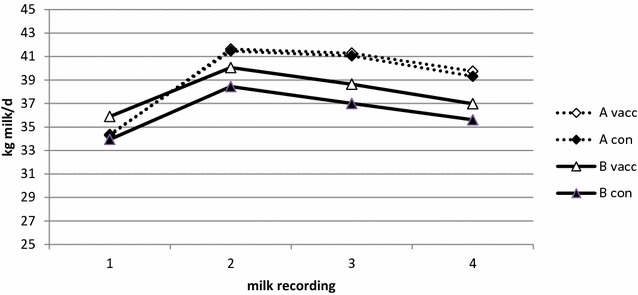
Milk production in vaccinated and not vaccinated cows. Milk production (kg milk/day; arithmetic average) at the first 4 monthly milk recordings after calving in vaccinated (vacc) and not vaccinated control (con) cows in herd A and B

The SCC (geometric average) and milk production (arithmetic average) at the first four milk recordings after calving per herd and treatment are presented in Figs. 1 and 2, respectively. There were no significant differences between vaccinated and unvaccinated cows for SCC or milk production. The predicted SCC was 67,200 cells/ml (95 % CI 56,300–80,200) in the vaccinated group and 65,700 cells/ml (95 % CI 55,200–78,200) in the control group (p = 0.77). The predicted milk production was 39.8 kg (95 % CI 38.9–40.7) in the vaccinated group and 39.6 kg (95 % CI 38.7–40.5) in the control group (p = 0.69).

### Prevalence of SCM and new SCM

The average prevalence of SCM cows at the first four milk recordings after calving among vaccinated cows was 42.1 % in herd A and 33.6 % in herd B. Corresponding numbers for unvaccinated cows were 40.5 and 36.9 %. There was no significant difference between treatment groups, the odds ratio (OR) for the vaccinated group compared to the control group was 0.96 (95 % CI 0.70–1.32; p = 0.79).

Among vaccinated cows not having SCM before drying off (also including primiparous cows), and having SCC data at all four milk recordings, new SCM was found in 69 (38.6 %) and 28 (27.2 %) cows in herd A and B, respectively, during the follow-up period. Corresponding numbers for unvaccinated cows were 63 (36.2 %) in herd A and 41 (33.9 %) in herd B. There were no significant differences between treatment groups (p = 0.75).

### Incidence of infectious mastitis

**Fig. 3:**
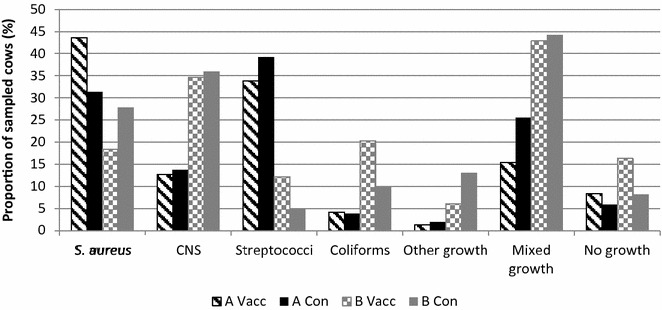
Presence of udder pathogens in vaccinated and not vaccinated cows. Proportions (%) of sampled cows having growth of different groups of udder pathogens (*CNS* coagulase-negative staphylococci) in 295 udder quarter milk samples from 232 cows with clinical or subclinical mastitis during the first 4 months after calving in vaccinated (vacc) and not vaccinated control (con) cows in herd A and B

A total of 295 udder quarter samples from 232 cows (71 vaccinated and 51 control cows in herd A, and 49 vaccinated and 61 control cows in herd B) with CM or SCM were analyzed for bacterial growth. Of those 232 cows, 172 (74.1 %) had growth of *S. aureus*, CNS, streptococci or coliforms. The proportions of sampled cows with growth of different groups of udder pathogens divided by herd and treatment group are shown in Fig. 3. The probability of mastitis due to *S. aureus*, CNS, streptococci or coliforms did not differ significantly between treatment groups. The OR for the vaccinated group compared to the control group was 1.03 (95 % CI 0.57–1.86, p = 0.93) for *S. aureus*, 0.97 (95 % CI 0.51–1.85, p = 0.92) for CNS, 1.09 (95 % CI 0.56–2.12, p = 0.81) for streptococci, and 2.00 (95 % CI 0.77–5.21, p = 0.15) for coliforms.

### Survival

Overall, the number of cows culled due to udder disease in the control group was 32 [8.1 %; 24 (10 %) in herd A and 8 (5.2 %) in herd B] during the follow-up period. The corresponding numbers for the vaccinated group was 37 [9.9 %; 34 (13.9 %) in herd A and 3 (2.3 %) in herd B]. No significant differences were found between treatment groups, the hazard ratio (HR) was 1.13 (95 % CI 0.7–1.82, p = 0.62).

## Discussion

According to the results of this study, no significant effects of vaccination were found on udder health parameters measured, and milk production, up to 4 months after calving, or on survival throughout lactation, on the two herds included in the study.

The results are partly in line with a study on commercial herds in UK [10], and in line with preliminary data from two studies in commercial herds in Iceland and Estonia [13, 14]. However, Bradley et al. [10] found that vaccinated cows were less likely to have severe CM and produced more milk than unvaccinated cows in the investigated herds. According to the authors, the vaccine effect was probably the result of the *E. coli* J5 component in the vaccine as most clinical cases were due to *E. coli* or *Streptococcus uberis*. In contrast, Schukken et al. [9] found a reduced duration of *S. aureus* IMI in vaccinated cows, and that vaccine efficacy was better in primiparous cows than in older cows. Vaccine effects on SCC, milk yield and survival, which are important economic incentives for the farmers, were, however, not presented in that study.

A relevant objection to the study is that not all cows were vaccinated as the randomly selected and equally sized control group remained in the herds, which could have resulted in a reduced likelihood for vaccine efficacy. To eliminate bias of this kind, a similar study in two North American dairy herds started by vaccinating all cows until 50 % of the herd was vaccinated, then only cows with even ear tag numbers were vaccinated [9]. Such an approach was not possible in the present study. It may be argued that some positive outcomes would have been expected anyhow as approximately half of the cows were vaccinated. If more than half of the cows must be vaccinated to achieve effect, the initial costs for introduction of a vaccination program increases. Based on experiences in the study the vaccine costs and benefits for a 100 cow herd was calculated. The yearly cost was estimated to be 45 €/cow based on extra work, and vaccine costs. If the vaccination resulted in a reduction of the estimated bulk milk SCC (12-months) with 55,000 cells/ml or a reduced CM incidence of 17 % the benefit-costs would break even [15]. No indications of such effects were, however, found in the present study.

Another weakness was that the present study, as well as previous studies [9, 10], was not blinded. As the farmers knew which cows belonged to the vaccination or control groups, it may have affected their will to investigate and sample cows after calving. It is also possible that the varying time point for the first vaccination in herd A, and the deviation from the exact time intervals in the vaccination protocol done in both herds resulted in a reduced vaccination effect.

Half of the total costs for vaccination used in the calculation above consisted of extra labor needed for sorting and identification of animals. An alternative, and possibly less labor consuming, vaccination protocol is the so called rolling model when all animals are vaccinated every 3rd month after a basic two injection immunization routine. The pros with such a protocol is that animal identification and sorting is not needed, but drawbacks are a higher cost for vaccine and that all animals will not have the highest possible protection during the high risk period in early lactation. Bradley et al. [10] did not see any differences in the incidence or prevalence of CM or SCM between cows vaccinated using the label model or the rolling model, and control cows. A significantly higher milk yield compared to control cows was found for the label group but not for the rolling group.

## Conclusions

Vaccination with a commercial polyvalent vaccine did not have any beneficial effects on udder health, milk production or survival in two commercial dairy herds with mastitis problems due to *S. aureus*.
